# Microbiome-inspired solutions to save human and planetary health

**DOI:** 10.3389/fmicb.2026.1744452

**Published:** 2026-02-25

**Authors:** Gabriele Berg, Markus Antonietti, Dilfuza Egamberdieva, Lise Korsten, Wisnu Adi Wicaksono

**Affiliations:** 1 Institute of Environmental Biotechnology, Graz University of Technology, Graz, Austria; 2 Leibniz Institute for Agricultural Engineering and Bioeconomy (ATB), Potsdam, Germany; 3 Institute for Biochemistry and Biology, University of Potsdam, Potsdam, Germany; 4 Department of Colloid Chemistry, Max Planck Institute of Colloids and Interfaces, Potsdam, Germany; 5 School of Medical, Central Asian University, Tashkent, Uzbekistan; 6 Faculty of Biology, National University of Uzbekistan, Tashkent, Uzbekistan; 7 Department of Plant and Soil Sciences, University of Pretoria, Hatfield, South Africa; 8 Department of Science, Technology and Innovation - National Research Foundation Centre of Excellence in Food Security, Pretoria, South Africa

**Keywords:** Anthropocene, environmental microbiome, microbial diversity, human microbiome, microbiome restoration

## Abstract

Microbial communities are dynamic networks that regulate nutrient cycling, energy flow, and ecosystem stability, making microbial diversity essential to the health and resilience of all living organisms and ecosystems. However, Anthropocene-driven human activities have led to substantial losses of microbial diversity in environmental and host-associated microbiomes. Despite their critical role, microbiome is underrepresented in conservation and public health strategies, creating a knowledge and intervention gap. Emerging strategies based on microbiome approach offer promising avenues for restoring microbial diversity and enhancing Planetary Health. Achieving these goals requires coordinated global policies, interdisciplinary collaboration, and recognition of microbes as essential partners in sustaining life on Earth.

## 1 Introduction

Microbial diversity represents the most extensive form of biological variation on Earth. Recent advancements in high-throughput sequencing and bioinformatics have substantially improved our understanding of microbial diversity and their functional roles (Berg et al., 2020). Despite its vital ecological importance, microbial diversity is currently experiencing an unprecedented decline. In the Anthropocene, human activities, such as including industrial agriculture, pollution, urbanization, excessive antibiotic use, and unhealthy dietary patterns, have reduced microbial diversity across environmental and host-associated systems, contributing to ecosystem degradation, reduced resilience, and rising non-communicable diseases in humans (Blaser and Dominguez-Bello, 2025; Geisen et al., 2019; Ruff et al., 2020; Voolstra et al., 2024).

The decline in microbial diversity is a critical, yet often overlooked, aspect of the global biodiversity crisis, with significant implications for ecosystem stability and Planetary Health. Evidence suggests that microbiomes are dynamic and adaptable to environmental disturbances. This resilience presents opportunities for targeted interventions, yet current research remains fragmented across disciplines, and microbiome-based solutions are rarely integrated into broader environmental and public health frameworks. There is a need for a unified perspective that connects microbial diversity loss across environmental, animal, and human systems and evaluates emerging strategies for mitigation and restoration. In this review, we address this gap by integrating evidence across environmental, animal, and human microbiomes to examine the ecological and evolutionary importance of microbial diversity, identify major Anthropocene drivers of its decline, and evaluate emerging strategies for its preservation and restoration. By emphasizing the central role of microbe within the One Health framework, we underscore that protecting microbial diversity is fundamental to maintaining the resilience and sustainability of life on Earth.

## 2 What do we know about the origin and importance of microbial diversity on Earth?

Microorganisms were the first living organisms on Earth. They developed in the ocean around 3.8–4 billion years ago (Nisbet and Sleep, 2001). Early microbial communities, including anoxygenic *Bacteria* and *Archaea*, played fundamental roles in shaping Earth’s biogeochemical cycles, driving processes such as the oxidation of ferrous iron and reduced sulfur compounds that supported early respiration and photosynthesis (Canfield, 2005; Lyons et al., 2014). These metabolic innovations laid the foundation for oxygenic photosynthesis, which ultimately led to the Great Oxidation Event (∼2.4 billion years ago) and the establishment of aerobic life (Weber et al., 2006).

Over billions of years of evolution, microorganisms have diversified their genomes, and recent analyses of the tree of life highlight the dominance of bacterial diversification. The estimated number of prokaryotic species on Earth may be as high as 1 trillion (10^12^), with their total abundance likely ranging between 4 and 6 × 10^30^ cells globally (Locey and Lennon, 2016; Whitman et al., 1998). Remarkably, this high diversity has only been revealed in the last three decades due to new molecular high-throughput methods. High-throughput sequencing, exemplified by the Earth Microbiome Project, has revealed millions of operational taxonomic units (OTUs) across a wide range of habitats, highlighting both the scope of microbial diversity and its functional potential (Gibbons and Gilbert, 2015; Thompson et al., 2017). This diversity underpins critical ecosystem functions, including nutrient cycling, energy flow, and the maintenance of environmental conditions that support life. Microbial diversity is the key to health for all organisms as well as for ecosystems and the entire planet Earth.

All animals and plants host communities of associated microorganisms, collectively referred to as their microbiomes, that carry a significant role of microbes in the health and functioning of the host. The concept of metaorganisms (or holobionts) describes a host organism such as a plant, animal, or human together with its associated microbiota, functioning as an integrated ecological unit (Bordenstein and Theis, 2015). For example, plants, which emerged roughly 470 million years ago, established symbiotic relationships with microbes such as mycorrhizal fungi, critical for nutrient acquisition and adaptation to terrestrial environments (Parniske, 2008). Co-evolution has produced plant microbiomes of high diversity and functional specialization, which support germination, stress resilience, and secondary metabolism. Although most studies focus on crops, emerging data from mosses and native plants reveal extraordinary microbial diversity across native plant families (Satjarak et al., 2022; Thompson et al., 2017; Wicaksono et al., 2021). Groussin et al. (2017) demonstrated mammalian gut microbiomes are shaped by host evolution and diet, with phylogeny strongly influencing recently diverged microbes. This reflects long-term host–microbe co-evolution and links microbiome composition to evolutionary and ecological adaptation. Similarly, human evolution occurred in environments rich in microbial diversity, with gut microbiomes shaped by long-term co-evolution and environmental exposure (Sonnenburg and Sonnenburg, 2019a). Comparative studies show that modern industrialized populations have experienced substantial reductions in gut microbial diversity compared to ancestral and non-industrialized populations (Carter et al., 2023; Harrison et al., 2025; Rampelli et al., 2015; Wibowo et al., 2021). Strain-level variation in gut bacteria reflects human phylogeography and long-term host–microbe adaptation, with traits such as oxygen intolerance and reduced genomes indicating co-dependence (Suzuki et al., 2022). Together, these findings highlight that plants, animals and humans are closely linked to their microbiomes, which have been shaped by long-term co-evolution and environmental exposure. Disruption of these host–microbe relationships therefore threatens essential functions underlying host health, resilience, and adaptation, underscoring the need to conserve both environmental and host-associated microbial diversity.

## 3 What do we know about the loss of microbial diversity?

**FIGURE 1:**
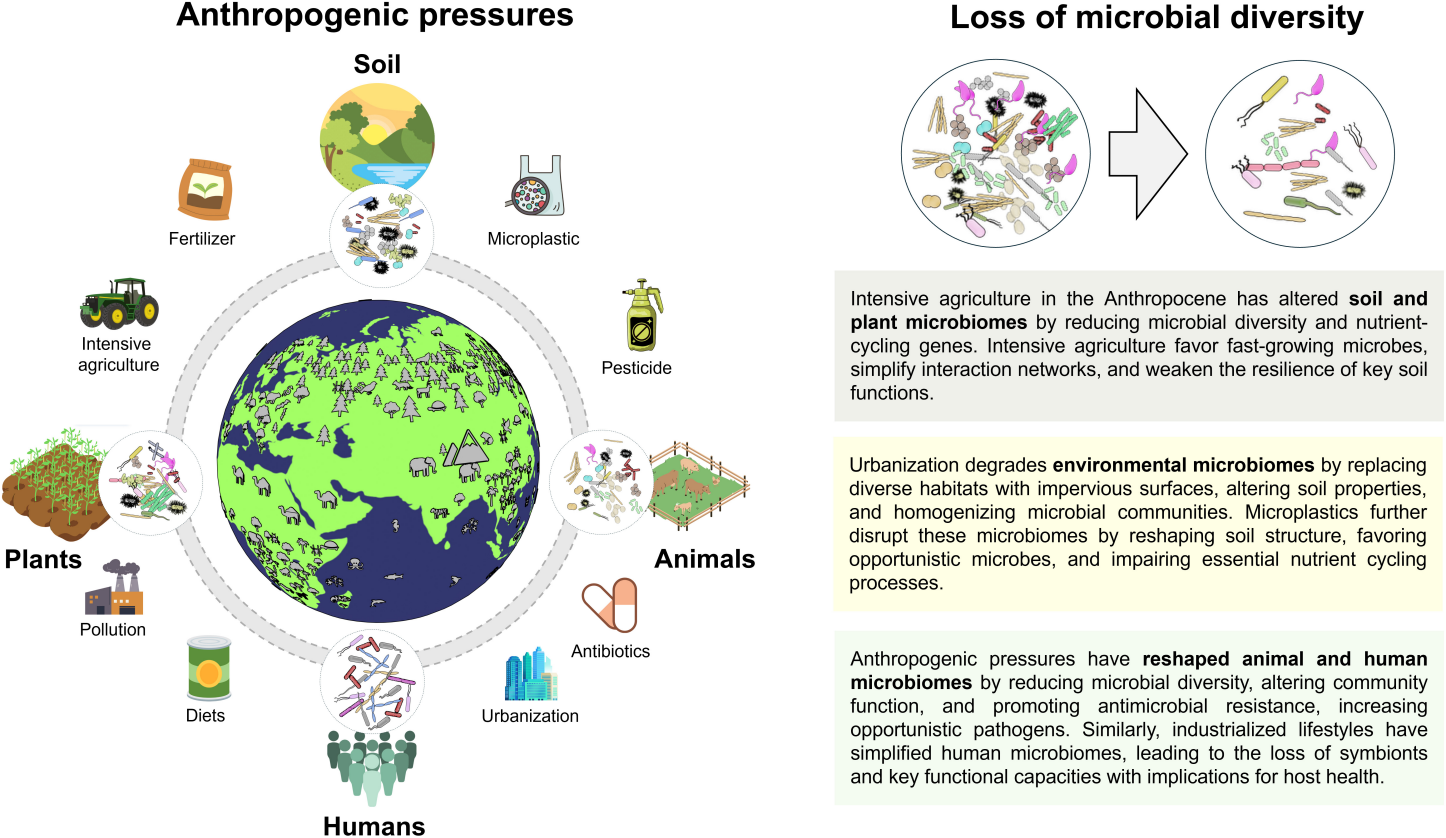
Schematic representation of the Anthropogenic pressures and its impact on environmental and host-associated microbiomes. This figure is created using Canva (https://www.canva.com) and BioRender (https://biorender.com).

The Anthropocene is used to describe the most recent period in Earth’s history when human activity started to have a significant impact on the planet’s climate and ecosystems. Due to the high global population and activities such as intensive agriculture and urbanization, we have long since exceeded the limits of Planetary Health, which poses a serious threat to our planet (Kitzmann et al., 2025; Richardson et al., 2023). These anthropogenic pressures also affect environmental and host-associated microbiomes, whose interconnections are increasingly recognized as critical for maintaining ecosystem functions and overall Planetary Health. In this section, we examine how these human-driven factors impact microbiomes across environments and hosts, and the implications for Planetary Health (Figure 1).

Environmental microbiomes, particularly in soils, have been profoundly altered by anthropogenic pressures associated with the Anthropocene. Intensive agricultural practices have emerged as a major driver of environmental microbiome alteration, profoundly affecting the composition, diversity, and functional capacity of soil microbial communities. Converting natural ecosystems to cropland causes ∼20% loss of microbial phylotypes and depletion of key nutrient-cycling genes, while intensive practices like high nitrogen fertilization further reduce bacterial and fungal diversity by ∼11% and ∼17%, highlighting the global impact of intensive agriculture on soil microbial communities (Peng et al., 2024; Yang et al., 2022). Repeated tillage, monocropping, and high inputs of synthetic fertilizers and pesticides disrupt soil structure and resource heterogeneity, favoring fast-growing, disturbance-tolerant microorganisms while suppressing slow-growing, functionally specialized taxa (Banerjee et al., 2019; Wang et al., 2025). Agrochemicals can directly inhibit symbiotic fungi and nitrogen-fixing bacteria (Fox et al., 2007), while excessive nutrient inputs reduce plant dependence on microbial mutualists (Beltran-Garcia et al., 2021), leading to their gradual loss. As microbial diversity declines, interaction networks become simplified, weakening functional redundancy and resilience in processes such as nitrogen cycling, phosphorus mobilization, and organic matter decomposition, ultimately compromising soil fertility and long-term plant productivity. Urbanization further contributes to environmental microbiome decline by replacing heterogeneous natural habitats with impervious surfaces and fragmented green spaces. It replaces diverse natural habitats with simplified, impermeable surfaces and fragmented green spaces, modifies soil physicochemical properties through compaction, pollution, nutrient enrichment, and microclimatic changes (Yang and Zhang, 2015). Urban expansion frequently results in the homogenization of microbial communities, with a shift toward increased abundance of fungal parasites, plant and human pathogens, and genes linked to greenhouse gas production, such as those involved in denitrification and methanogenesis, when compared to adjacent natural ecosystems (Delgado-Baquerizo et al., 2021).

Accumulation of pollutants from the Anthropocene, has severely impacted soil microbiomes and reduced the multifunctionality of ecosystems essential for environmental stability. For example, microplastics influence the physical structure of soil by altering porosity, aggregation, and water retention, which subsequently affects oxygen diffusion and the heterogeneity of microbial habitats (Nuñez et al., 2025; Rillig et al., 2024). Microplastics provide new surfaces for microbial colonization, forming the “plastisphere,” which is selectively enriched in bacteria and microbial eukaryotes, including potential pathogens, some of which carry antibiotic resistance genes (ARGs) and may promote shifts toward opportunistic microbial communities (Rillig et al., 2024). Moreover, at high concentrations of microplastics (5% w/w in soil), essential microbial processes, such as carbon decomposition, nitrogen cycling, and enzyme-mediated nutrient turnover, are disrupted, ultimately leading to a reduction in ecosystem multifunctionality (Liu et al., 2024).

At the same time, global land-use patterns reveal limited progress toward more sustainable agricultural practices. Although the area dedicated to organic farming increased substantially in 2021, organically managed cropland still represented just over 5% of total global agricultural land (FAO, 2025). This limited adoption is particularly concerning given that over 50% of the world’s population currently resides in urban areas, a figure projected to rise to nearly two-thirds by 2050, placing growing demands on food systems and surrounding ecosystem. Adding to these environmental challenges, annual microplastic emissions are estimated at 10–40 million tones, with projections indicating a possible doubling of this load by 2040 (Thompson et al., 2024). These widespread and consistent shifts underscore the pronounced sensitivity of environmental microbiomes to anthropogenic pressures and the current trend suggest that such alterations are likely to persist over time.

Anthropogenic pressures have also profoundly altered animal microbiomes, with consequences for host health and antimicrobial resistance. Antibiotic use in livestock, aquaculture, and wildlife disrupts gut microbial communities and promotes antibiotic resistance genes (ARGs), creating reservoirs of resistant bacteria. For example, poultry exposed to growth-promoting or therapeutic antibiotics show increased prevalence of ESBL *E. coli*, vancomycin-resistant *Enterococcus faecalis* and *E. faecium*, and fluoroquinolone-resistant *Campylobacter jejuni* (Gupta et al., 2021). Similarly, rehabilitated seals treated with antibiotics exhibit reduced gut microbial diversity and sustained ARG enrichment (Rubio-Garcia et al., 2025). Chemical pollutants, including heavy metals, pesticides, and microplastics also perturb the animal microbiomes. In livestock, microplastics interact with rumen microbiota, altering fermentation efficiency and host energy metabolism, which can affect nutrition and productivity (Eichinger et al., 2025). Environmentally relevant microplastic exposure in wild seabirds reduces commensal taxa while enriching zoonotic, antibiotic-resistant, and plastic-degrading microbes, reflecting shifts in community structure (Fackelmann et al., 2023). Habitat fragmentation, altered diets, and human contact further homogenize wildlife microbiomes with implications for host immunity and cross-species pathogen transmission (Fackelmann et al., 2021; Ingala et al., 2019). For example, generalist rodents (*Proechimys semispinosus*) in human-impacted areas harbor microbiota enriched in taxa associated with domesticated animals and potential pathogens (Fackelmann et al., 2021). Together, these Anthropocene drivers such as. antibiotics, microplastics, and habitat alteration, are reshaping animal microbiomes by reducing microbial diversity and function and promoting the spread of antimicrobial resistance, with cascading effects on host health, and cross-species pathogen transmission.

Parallel to changes observed in environmental and animal systems, industrialization and modern lifestyles have substantially reshaped human-associated microbiomes. Our current understanding suggests that the microbiota of individuals in industrialized societies is influenced by recent developments in medicine, diet, sanitation, and food processing (Sonnenburg and Sonnenburg, 2019b). For example, studies across urbanization gradients within two provinces of China showed reduced gut microbial diversity in urban populations. Moreover, a total of 26 previously undetected OTUs absent and 70 OTUs less abundant compared to rural populations, indicating loss and suppression of novel human symbionts during urbanization (Sun et al., 2022). Comparative studies have also consistently shown that traditional populations retain microbial taxa that are either diminished or absent in industrialized populations (Carter et al., 2023; Harrison et al., 2025; Wibowo et al., 2021). However, the divergence is not solely taxonomic; it also encompasses functional capacities of the microbiome. For instance, the Hadza of Tanzania, a hunter-gatherer society, exhibits gut microbiota enriched in carbohydrate-active enzymes (CAZymes) compared to industrialized populations (Rampelli et al., 2015), reflecting a broader metabolic capacity for degrading complex polysaccharides. One of the characteristic symbiont taxa absent in urban-industrialized societies is *Treponema*, a known carbohydrate-metabolizing microorganism (Obregon-Tito et al., 2015). Analysis of palaeofaeces and non-industrialized samples versus industrialized ones shows that starch- and glycogen-degrading CAZymes are enriched in ancient and traditional gut microbiomes (Wibowo et al., 2021). In contrast, mucin- and alginate-degrading CAZymes, which break down the protective mucus layer of the gut for energy, are more prevalent in industrialized populations suggesting a shift toward microbes that utilize host-derived substrates in the absence of dietary fiber. The composition, functional redundancy, and diversity of the microbiome likely reflect long-standing evolutionary adaptations to diverse, fiber-rich diets, in contrast to the processed and fiber-poor diets prevalent in industrialized settings.

The loss of microbial diversity is increasingly recognized as a consequence of broader declines in environmental microbial diversity and reduced human–environment microbial exchange. Simultaneously, there has been a global surge in non-communicable diseases (NCDs) as the world’s leading causes of death (The World Bank, 2024). While causality between microbiota changes and NCD pathogenesis remains under investigation, emerging evidence indicates a significant association between the development of the human gut microbiome, particularly in cases of dysbiosis, which involves disruption of the structure and function of the gut microbial community, and disease outcomes such as obesity, type 1 diabetes, inflammatory bowel disease, and asthma (Depner et al., 2020; Gao et al., 2018; Halfvarson et al., 2017; Vatanen et al., 2016). Limited contact with soils, natural ecosystems, and diverse environmental microbiota, especially in urban, highly sanitized environments, restricts exposure to microbes that are important for microbiome development and immune system maturation. Exposure to biodiverse soils and environmental microbes has been shown experimentally to modulate gut and skin microbiota composition and enhance anti-inflammatory immune markers (e.g., higher regulatory cytokines) in human intervention studies (Roslund et al., 2022; Saarenpää et al., 2024). In this context, urbanization and the progressive disconnection from natural microbial reservoirs represent a key mechanistic link connecting environmental microbiome to the rising prevalence of non-communicable diseases in industrialized populations.

## 4 Lessons learned from the most significant man-made environmental disasters of the last century: desiccation of the Aral Sea

The desertification of the Aral Sea basin, located in Uzbekistan and Kazakhstan, constitutes one of the most severe anthropogenic environmental disasters of the 20th century. Since the 1960s, this once-vast inland water body, the fourth largest in the world, has undergone dramatic shrinkage. Between 1960 and 2018, the Aral Sea in Central Asia underwent a significant reduction of approximately 88% in its surface area and lost over 1,000 km^3^ of water volume. This environmental decline was primarily driven by human activities, including extensive irrigation and river diversion (Yang et al., 2020). This has led to an extreme increase in salinity levels and the accumulation of hazardous substances, including carcinogens and heavy metals, within the exposed and desiccated basin (Jensen et al., 1997; Micklin, 2010; Zetterström, 1999).

The ongoing desiccation of the Aral Sea presents a unique natural laboratory due to its extreme environmental conditions such as accumulation of toxin and high salinity, which enables the study of microbial community dynamics and functional adaptation to environmental change. However, the microbial exploration of the Aral Sea has only recently been done. In the Aral Sea basin, bacterial communities respond to extreme stressors, including elevated salinity, heavy metal contamination, and progressive desertification (Shurigin et al., 2019). It is not surprising that the drastic environmental changes in the region have selected for extremophilic and halophilic microbial taxa capable of surviving in hypersaline and oligotrophic conditions (Chernyh et al., 2024; Shurigin et al., 2019). Using the chronosequence approach, recent findings demonstrate that rhizosphere assembly through the selective filtering of microbial taxa with specific functional traits serves as a key mechanism facilitate plant survival under extreme conditions in the Aral sea (Wicaksono et al., 2022). Metagenome-assembled genomes revealed core functions, such as osmoprotectant production and nitrate reduction, consistently present in both archaea and bacteria. However, these taxa were recruited at different stages, indicating functional redundancy between the two domains. This suggests that the plant actively adapts to its environment by recruiting beneficial microbes that support its growth and stress tolerance. Bacteria were not the only microbiome component undergoing shifts. *In silico* analyses revealed that viruses were associated with dominant prokaryotic hosts such as *Gammaproteobacteria*, *Actinomycetia*, and *Bacilli*. A lysogenic lifestyle was predominant in areas desiccated for 5 years, corresponding to the early stages of revegetation. Predicted viral auxiliary metabolic genes (AMGs) indicate potential roles in biofilm formation, stress tolerance, and nutrient cycling (Wicaksono et al., 2023). These findings highlight the often-overlooked ecological significance of bacterial-viral interactions in terrestrial ecosystems, especially during natural revegetation processes.

The Aral Sea serves as a compelling example of microbial resilience and adaptability, offering a source of extremophilic traits and ecological functions with direct relevance to restoration ecology and climate-resilient land management. These findings illustrate several key principles for microbiome-based restoration. First, targeted recruitment of functional microbial taxa i.e., confer stress tolerance and maintain nutrient acquisition by plants highlights the potential to enhance ecosystem resilience through microbiome based approach. Second, functional redundancy stabilizes critical ecosystem processes, indicating that preserving or restoring overlapping microbial functions may buffer ecosystems against environmental stresses. Third, multi-kingdom interactions including bacteria, archaea, and viruses, play integral roles in adaptation, suggesting that restoration strategies should consider the broader microbial network rather than focusing solely on bacteria. Fourth, extremophilic and functionally specialized taxa from the Aral Sea may serve as candidate inoculants or bioindicators for rehabilitating degraded or salinized soils. Monitoring shifts in microbial composition, functional potential, and gene expression provides early-warning signals for ecosystem recovery and allows assessment of restoration effectiveness. These findings suggest that microbiome-based approaches targeting functional traits, ensuring redundancy, and considering microbial networks, potentially improve the effectiveness of ecosystem recovery in degraded landscapes.

## 5 What can we do to preserve and restore microbial diversity?

The soil, plant, and human microbiomes are intricately interconnected. The soil microbiome plays a critical role in supporting plant development by facilitating nutrient cycling, enhancing stress tolerance, and shaping the composition of the plant-associated microbiota. In turn, plants modulate soil microbial communities through the release of root exudates and the deposition of organic matter. These bidirectional interactions extend to the human domain via the edible plant microbiome, refers to the communities of microbes, such as bacteria and fungi, that naturally colonize the tissues of plants we eat, especially when consumed raw, which can influence the human microbiome through dietary intake and environmental exposure (Berg et al., 2025). This continuum highlights how soil microbial diversity impacts food quality, nutritional content, and ultimately, the structure and function of the human gut microbiome.

**FIGURE 2:**
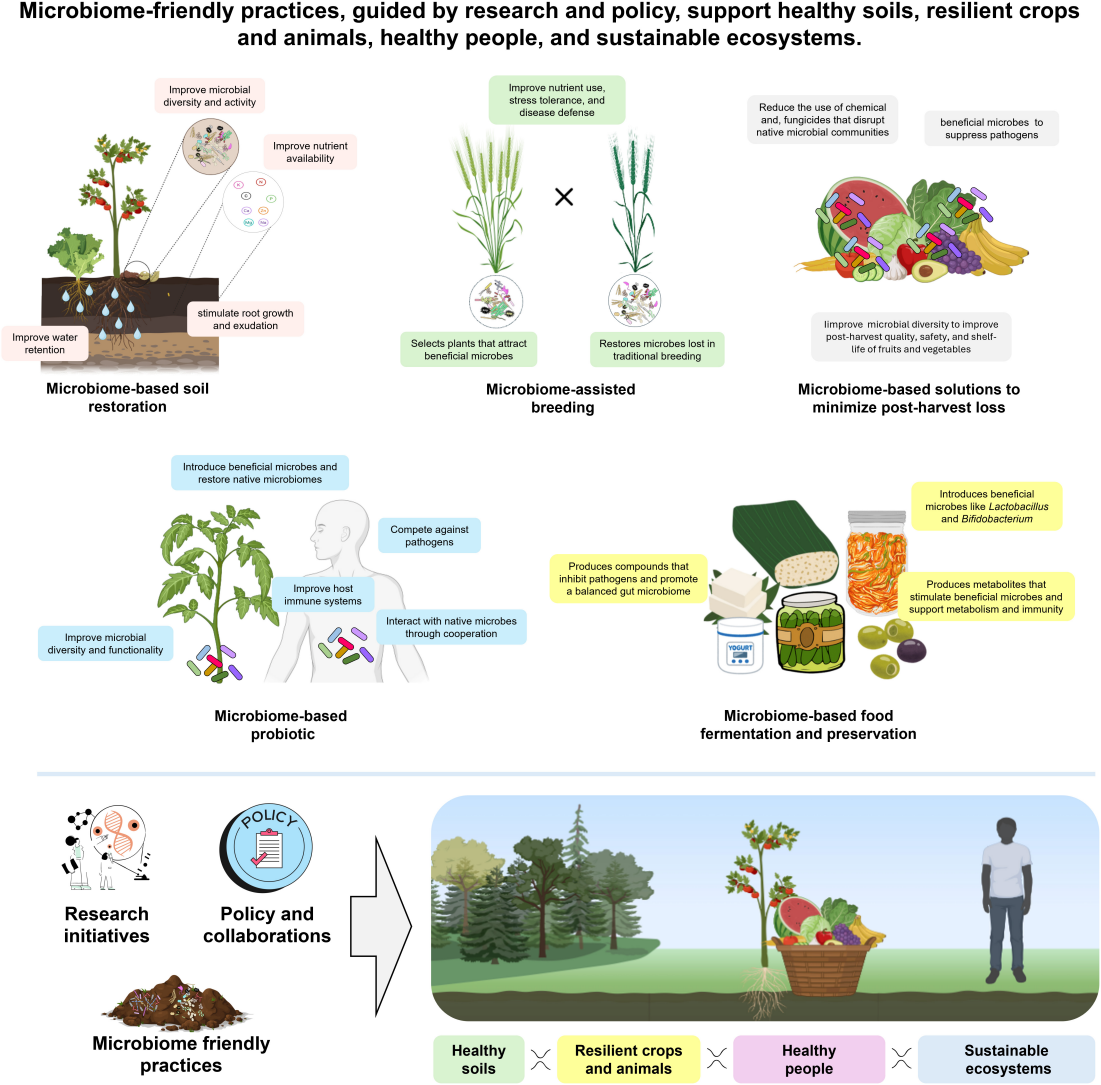
Microbiome-based solutions aim to preserve and restore microbial diversity. This figure was created using Canva (https://www.canva.com) and BioRender (https://biorender.com).

However, in the Anthropocene, human activities, such as industrial agriculture, urbanization, and widespread antimicrobial use, have contributed to a global decline in microbial diversity. Addressing these challenges requires integrated, microbiome-based solutions to restore ecological balance. Microbiome-based solutions encompass broad strategies designed to maintain or restore microbial diversity and functionality in soils, plants, animals, and humans. These solutions are implemented through specific interventions, including ecological restoration, microbiome-based agricultural practices, probiotics and policy interventions. Targeted interventions, such as probiotics, biofertilizers, or bioremediation, can harness these functional capacities to modulate microbial networks, enhancing host health, ecosystem function, and environmental sustainability (Kamble et al., 2024). By linking solutions to actionable interventions, this framework highlights how microbiome-centered approaches can enhance ecosystem resilience, host health, and Planetary Health. Here, we discuss microbiome-based solutions to preserve and restore microbial diversity (Figure 2).

### 5.1 Microbiome-based soil restoration

Soils play a central role in terrestrial ecosystems. However, this resource is under threat worldwide: 60% of soils in Europe are already damaged; worldwide, 33% are degraded (Smith et al., 2024). Microbiome-based soil restoration uses amendments to enhance soil health and rehabilitate degraded soils by supporting natural processes such as nutrient cycling, soil structure formation, and pollutant degradation. By supporting microbial activity and creating favorable conditions for diverse microbial communities, it promotes resilient, functional, and sustainable soil ecosystems. For instance, biochar amendments improve soil pH, organic matter, and nutrient availability, while providing heterogeneous surface microenvironments and diversified ecological niches that support a functional soil microbiome (Lin et al., 2025). Organic amendments, such as compost supply labile carbon and nutrients that increase resource heterogeneity, supporting diverse microbial communities and enhancing both taxonomic richness and functional redundancy in the soil microbiome (Shu et al., 2022). Another key example is humic acid; an organic substance derived from the decomposition of plant and animal residues. Natural humic acid as well as innovative, artificially produced humic substances produced within the bioeconomy circle contributes to soil aggregation, increases water retention, and improves nutrient availability, factors that collectively create a more favorable environment for the growth and activity of beneficial soil microbes (Yang et al., 2021). Additionally, its ability to bind heavy metals and toxic compounds aids in microbial-mediated remediation, helping to detoxify soils. Humic-based products can also stimulate root growth and exudation, indirectly shaping the composition and activity of the soil microbiome (Lumactud et al., 2022). By enhancing microbial diversity and activity, microbiome-based amendments provide a sustainable strategy to restore soil health, improve ecosystem functions,

### 5.2 Microbiome-based probiotics

Microbiome-based probiotics refer to the strategic introduction of beneficial microorganisms to support and improve the native microbiomes of soil, plants, and humans. In agricultural systems, applying probiotic bacterial consortia can beneficially reshape the rhizosphere microbiome, leading to enhanced plant growth by modifying both the composition and functionality of native microbial communities (Francioli et al., 2025; Hu et al., 2021). These outcomes are often achieved through multifunctional bioinoculants that possess capabilities such as nitrogen fixation, phosphorus solubilization, siderophore production, and the synthesis of phytohormones like auxins and gibberellins (Egamberdieva et al., 2019). Moreover, these inoculants can activate systemic resistance in plants and induce microbiome shifts, improving their resilience against pathogens and environmental stressors. Importantly, in systems where intensive agriculture has diminished microbial diversity, the use of such microbial inocula offers a promising strategy to restore both soil and plant microbial diversity. Similarly, in humans, successful human probiotic consortia are defined by traits that enable health benefits [reviewed in Latif et al. (2023)]. Functional complementarity allows strains to produce short-chain fatty acids, vitamins, antimicrobials, and immunomodulatory molecules, while ecological resilience ensures survival through gastric acidity and bile. Effective probiotics interact with the native microbiome and host via mucosal adhesion, pathogen exclusion, and immune modulation, supporting microbial diversity recovery after disturbances like antibiotic treatment (FitzGerald et al., 2022; Latif et al., 2023). Across different hosts and environments, successful probiotics rely on functional complementarity, where introduced strains occupy different ecological niches, and dynamic interactions with native microbes through competition, cooperation, and signaling. Moreover, environmental filtering, like soil chemistry or gut conditions, also shapes which strains establish. These shared principles in designing probiotics, focusing on diversity, functional synergy, and compatibility, can be applied both to agricultural and clinical settings.

### 5.3 Microbiome-assisted breeding

Domestication, intensified by breeding programs, has focused on selecting higher-yielding plant genotypes. The altered microbiome resulting from domestication often provides less benefit to the plant [reviewed in Nerva et al. (2022)]. For example, domesticated rice varieties alter the rhizosphere microbiome, reducing nitrogen-fixing taxa and increasing nitrous oxide emissions, highlighting the ecological consequences of microbiome shifts induced by breeding (Chang et al., 2025). Similarly, a maize germplasm chronosequence demonstrated that historical breeding practices influence the recruitment of rhizosphere microbiota, with older landraces hosting more diverse and functionally beneficial microbial communities than modern cultivars (Favela et al., 2021). This is likely because modern crop cultivars may have lost certain genetic traits essential for recruiting host-specific microbiota, traits that are still present in their wild relatives. A similar phenomenon can be observed in humans, where industrialization has also impacted the composition and function of the native microbiome. Microbiome-assisted breeding is an innovative strategy that incorporates the plant’s associated microbial communities into conventional breeding efforts to improve crop productivity, resilience, and sustainability. Instead of focusing solely on plant genetics, this approach selects for plants that effectively recruit and sustain beneficial microbes, enhancing nutrient absorption, stress resistance, and disease protection. This enrichment increases both taxonomic and functional diversity within the plant microbiome. Recent genomic studies provide further support for this approach. Deng et al. (2021) identified plant loci associated with the heritability of rhizosphere microbiomes, showing that specific host genes strongly influence microbial community composition. Yang et al. (2024) demonstrated that integrating microbiome-enabled genomic selection significantly improves the prediction accuracy of nitrogen-related traits in maize, illustrating the potential of combining host genetics and microbial recruitment for trait improvement. Guilherme Pereira et al. (2025) demonstrated that breeding for microbiomes can confer specific functional traits, such as salt tolerance, indicating that targeted microbial recruitment can be integrated into breeding programs to improve plant resilience. A recent study highlighted the concept of “*Microbiome genes*” (M-genes), defined as a group of host plant genes that influence and shape the composition and function of the plant’s associated microbial communities (Cernava, 2024; Su et al., 2024). Host genes, such as *OsPAL02* in rice, regulate microbiome recruitment by controlling the production of lignin-precursor metabolites like 4-hydroxycinnamic acid, which selectively enrich beneficial (United Nations Environment Programme, 2024) microbes and maintain phyllosphere community homeostasis. Identifying which of these genes have been lost or altered during breeding, and which control microbial recruitment, represents a crucial first step toward integrating the microbial component into breeding programs to enhance plant phenotypes. Together, these findings underscore the concept that plant breeding can be designed to enhance microbial diversity and ecosystem function.

### 5.4 Microbiome-based solutions to minimize post-harvest loss

Post-harvest food loss represents a major economic and environmental challenge, with around 13% of global food lost along the supply chain before reaching retail. This loss contributes significantly to food waste, greenhouse gas emissions, and inefficient use of agricultural land (United Nations Environment Programme, 2024). Much of this loss results from microbial spoilage, fungal infections, and pest damage that compromise the quality and safety of fresh produce. However, conventional post-harvest treatments, such as the use of strong chemical washes, prolonged refrigeration, and certain packaging techniques, can disrupt fresh produce native microbial populations, potentially diminishing their beneficial impact on human health. Microbiome-derived technologies provide innovative and sustainable methods to tackle food loss during the post-harvest stage, a critical period when a significant portion of agricultural products spoil, decay, or become contaminated. By utilizing beneficial microbes as natural biocontrol agents, these microbiome-based solutions can suppress harmful pathogens [reviewed in Droby and Wisniewski (2018), Kusstatscher et al. (2020), Wassermann et al. (2022)]. This approach can also potentially reduce reliance on conventional chemical treatments, such as fungicides and disinfectants, which often indiscriminately kill both harmful and beneficial microbes. Higher microbial diversity is generally associated with greater resilience and defense against spoilage and disease. By minimizing chemical exposure, the native microbial communities on fruits and vegetables are preserved, allowing beneficial taxa to persist and interact. These interactions help maintain a more diverse post-harvest microbiome, which can contribute to improved post-harvest quality and safety.

### 5.5 Microbiome-based food fermentation and preservation

Fermentation preserves food by using microorganisms to break down sugars and starches to increase digestibility, nutrient production and absorption and to enrich probiotic bacteria. Additionally, fermented foods are linked to a reduced risk of certain non-communicable diseases. Interestingly, humans have been consuming fermented food since ancient times, with the earliest evidence dating to around 7,000 BC in China. Although it has been used very successfully for thousands of years, it has been almost forgotten in industrial food production, especially in Europe. The key difference here is that we now have a better understanding and appreciation of its health-promoting properties and microbial diversity. Fermented foods can improve gut microbial diversity through several mechanisms. First, fermented foods contain diverse microbial communities that can support gastrointestinal health and influence immune function, like the effects of probiotics (Marco et al., 2017). Regular consumption of traditionally fermented products such as yogurt, kefir, kimchi, and sauerkraut introduces diverse microbial taxa, including *Lactobacillus* and *Bifidobacterium* species, which are often depleted in individuals with reduced microbial diversity (Ibrahim et al., 2023). Second, fermentation generates bioactive metabolites, such as short-chain fatty acids (SCFAs), bioactive peptides, and polyphenol derivatives, which selectively stimulate the growth of commensal microbes and improve metabolic (e.g., anti-obesogenic, antidiabetic) and immune (e.g., anti-inflammatory) functions (Mukherjee et al., 2024; Wei et al., 2025). Third, fermented foods produce bioactive compounds, including bacteriocins and polyphenols, that selectively inhibit opportunistic or pathogenic bacteria while supporting the growth of beneficial gut microbes, thereby promoting a more balanced and diverse gut microbiome (Mukherjee et al., 2024).

## 6 Policy and collaborative strategies for microbial diversity conservation

Protecting microbial diversity demands not only rigorous research but also the development of coordinated policy frameworks and collaboration across local, national, and global levels. Increasing awareness of the critical role played by the soil microbiome is essential. Several organizations have taken steps in this direction. For instance, the Global Soil Partnership (GSP), managed by the Food and Agriculture Organization (FAO), acknowledges microbial diversity as a key element of soil health and advocates for sustainable land management policies that support the preservation of soil microbiomes (Rodríguez Eugenio, 2021). Similarly, the Convention on Biological Diversity (CBD), which establishes global biodiversity targets, has recently started to emphasize the significance of microbial diversity. Additionally, international research initiatives such as the Earth Microbiome Project (Thompson et al., 2017) and the Human Microbiome Project (Turnbaugh et al., 2007) provide essential data that can support the development of evidence-based policies. For example, a global microbiome survey revealed that more than 70% of areas with predicted high microbial richness are currently not protected (Guerra et al., 2022; Van Nuland et al., 2025), highlighting the urgent need to integrate microbial hotspots into conservation strategies.

Well-designed policies are needed to encourage sustainable land management, and promote practices that nurture healthy microbiomes in soil, plants, and humans. Furthermore, joint initiatives involving multi stakeholders - governments, researchers, farmers, industries, and communities are crucial to increase awareness, facilitate knowledge exchange, and implement effective strategies for preserving and restoring microbial diversity. Recognizing this critical gap, the International Union for Conservation of Nature (IUCN) recently established the Microbial Conservation Specialist Group, a major global initiative dedicated to protecting microbial biodiversity (Gilbert et al., 2025). This group will form a worldwide network of experts, including representatives from low- and middle-income countries as well as Indigenous communities, to provide guidance on conservation targets and develop a framework for evaluating conservation priorities. Comprising microbiologists, ecologists, traditional knowledge holders, and conservation leaders, the group aims to integrate microbial conservation into broader environmental policies, advance research on the ecological roles of microbes, and raise awareness of the vital functions microbes perform in ecosystems. Educating and engaging policymakers, stakeholders, and the public is therefore essential to ensure strategies for preserving and restoring microbial diversity are effectively supported, implemented, and prioritized alongside broader environmental and public health initiatives.

## 7 Concluding remarks

Microbial diversity serves as a fundamental foundation for the health and resilience of soils, plants, humans, and entire ecosystems. The microbiome-based strategies discussed, ranging from soil restoration and probiotics to assisted breeding and post-harvest interventions, present promising and sustainable opportunities to improve environmental health, and ensure food security. Yet, unlocking the full benefits of these approaches requires more than scientific breakthroughs; it calls for coordinated policies, cross-sector collaboration, and a global commitment to protecting and fostering microbial diversity. Raising awareness and embedding microbial conservation within broader environmental policies will be essential to preserving this unseen but important contributors to One and Planetary Health.
