# Correction: Microbiome-inspired solutions to save human and planetary health

**DOI:** 10.3389/fmicb.2026.1831892

**Published:** 2026-04-02

**Authors:** Gabriele Berg, Markus Antonietti, Dilfuza Egamberdieva, Lise Korsten, Wisnu Adi Wicaksono

**Affiliations:** 1 Institute of Environmental Biotechnology, Graz University of Technology, Graz, Austria; 2 Leibniz Institute for Agricultural Engineering and Bioeconomy (ATB), Potsdam, Germany; 3 Institute for Biochemistry and Biology, University of Potsdam, Potsdam, Germany; 4 Department of Colloid Chemistry, Max Planck Institute of Colloids and Interfaces, Potsdam, Germany; 5 School of Medical, Central Asian University, Tashkent, Uzbekistan; 6 Faculty of Biology, National University of Uzbekistan, Tashkent, Uzbekistan; 7 Department of Plant and Soil Sciences, University of Pretoria, Hatfield, South Africa; 8 Department of Science, Technology and Innovation - National Research Foundation Centre of Excellence in Food Security, Pretoria, South Africa

**Keywords:** Anthropocene, environmental microbiome, microbial diversity, human microbiome, microbiome restoration

Affiliation [Department of Plant and Soil Sciences, University of Pretoria, Hatfield, South Africa] was omitted for author [Lise Korsten].

Author [Lise Korsten] was erroneously assigned to affiliation [Medical School, Central Asian University, Tashkent, Uzbekistan]. This affiliation has now been removed for author [Lise Korsten].

The correct affiliation reads:

Gabriele Berg^1, 2, 3, 4*^, Markus Antonietti^4^, Dilfuza Egamberdieva^5, 6^, Lise Korsten^7, 8^, Wisnu Adi Wicaksono^1*^

^1^Institute of Environmental Biotechnology, Graz University of Technology, Graz, Austria

^2^Leibniz Institute for Agricultural Engineering and Bioeconomy (ATB), Potsdam, Germany

^3^Institute for Biochemistry and Biology, University of Potsdam, Potsdam, Germany

^4^Department of Colloid Chemistry, Max Planck Institute of Colloids and Interfaces, Potsdam, Germany

^5^School of Medical, Central Asian University, Tashkent, Uzbekistan

^6^Faculty of Biology, National University of Uzbekistan, Tashkent, Uzbekistan

^7^Department of Plant and Soil Sciences, University of Pretoria, Hatfield, South Africa

^8^Department of Science, Technology and Innovation - National Research Foundation Centre of Excellence in Food Security, Pretoria, South Africa

An incorrect **Funding** statement was provided. The correct funder is [TU Graz Open Access Publishing Fund]/the correct **Funding** statement reads:

The author(s) declared that financial support was received for this work and/or its publication. This research project received support from the Eurasia-Pacific Uninet (EPU 33 2023). Open access funding was provided by the TU Graz Open Access Publishing Fund.

**Acknowledgments**

In the acknowledgments, [The TU Graz Open Access Publishing Fund that supported the Open Access Publication Fund] was not included.

The current statements reads:

We greatly appreciate the help of Matthias Schweitzer (Graz University of Technology), Austria, in the preparation of the figure elements. Open access funding was supported by the TU Graz Open Access Publishing Fund.

The original version of this article has been updated.

